# Identification of an Immune-Related Long Noncoding RNA Pairs Model to Predict Survival and Immune Features in Gastric Cancer

**DOI:** 10.3389/fcell.2021.726716

**Published:** 2021-09-21

**Authors:** Shenglei Song, Shuhao Liu, Zhewei Wei, Xinghan Jin, Deli Mao, Yulong He, Bo Li, Changhua Zhang

**Affiliations:** ^1^Digestive Diseases Center, The Seventh Affiliated Hospital of Sun Yat-sen University, Shenzhen, China; ^2^Department of Gastrointestinal Surgery, The First Affiliated Hospital of Sun Yat-sen University, Guangzhou, China; ^3^Guangdong Provincial Key Laboratory of Digestive Cancer Research, The Seventh Affiliated Hospital of Sun Yat-sen University, Shenzhen, China; ^4^Scientific Research Center, The Seventh Affiliated Hospital of Sun Yat-sen University, Shenzhen, China

**Keywords:** gastric cancer, long noncoding RNA, immune signature, prognosis, riskScore

## Abstract

**Background:** Gastric cancer (GC) remains one of the most malignant tumors around the world, and an accurate model that reliably predicts survival and therapeutic efficacy is urgently needed. As a novel predictor for prognosis in a variety of cancers, immune-related long noncoding RNA pairs (IRlncRNAPs) have been reported to predict tumor prognosis. Herein, we integrated an IRlncRNAPs model to predict the clinical outcome, immune features, and chemotherapeutic efficacy of GC.

**Methods:** Based on the GC data obtained from The Cancer Genome Atlas (TCGA) database and the Immunology Database and Analysis Portal (ImmPort), differentially expressed immune-related long noncoding RNAs (DEIRlncRNAs) were identified. Least absolute shrinkage and selection operator (LASSO) regression and Cox regression analysis were used to select the most appropriate overall survival (OS)-related IRlncRNAPs to develop a prognostic signature. The riskScore of each sample was calculated by comparing the long noncoding RNA expression level in each IRlncRNAP. Based on the riskScore for each patient, GC patients were divided into high- and low-risk groups. Then, the correlation of the signature and riskScore with OS, clinical features, immune cell infiltration, immune-related gene (IRG) expression and chemotherapeutic efficacy in GC was analyzed.

**Results:** A total of 107 DEIRlncRNAs were identified which formed 4297 IRlncRNAPs. Fifteen OS-related IRlncRNAPs were selected to develop a prognostic model. GC patients could be accurately classified into high- and low-risk groups according to the riskScore of the prognostic model. The 1-, 2-, 3-, and 5-year receiver operating characteristic (ROC) curves for the riskScore were drawn and the area under the curve (AUC) values were found to be 0.788, 0.810, 0.825, and 0.868, respectively, demonstrating a high sensitivity and accuracy of this prognostic signature. Moreover, the immune-related riskScore was an independent risk factor. Patients showed a poorer outcome within the high-risk group. In addition, the riskScore was found to be significantly correlated with the clinical features, immune infiltration status, IRG expression, and chemotherapeutic efficacy in GC.

**Conclusion:** The prognostic model of IRlncRNAPs offers great promise in predicting the prognosis, immune infiltration status, and chemotherapeutic efficacy in GC, which might be helpful for the selection of chemo- and immuno-therapy of GC.

## Introduction

Gastric cancer remains one of the most malignant tumors around the world. An estimated 1,089,103 (5.6%) new cases and 768,793 (7.7%) new cancer deaths were attributable to GC in 2020 (Sung et al., 2021). Though the incidence of GC has decreased in most populations as a result of the improvement of food storage conditions and the eradication of *Helicobacter pylori* (Howson et al., 1986), incidence and mortality rates are still high in Eastern Asia, Eastern and Central Europe, and Latin America (Balakrishnan et al., 2017). Treatment methods for GC primarily include surgery, radiotherapy, chemotherapy and molecular targeted therapy, and the prognosis of GC has improved remarkably. But the 5-year survival rate among advanced GC remains low, reported to be under 20% (Thomassen et al., 2014). In recent years, immunotherapy has become increasingly popular and has achieved great success in GC. Some studies have shown that immunotherapy significantly improves both overall survival (OS) and progression-free survival (PFS) in advanced GC (Fuchs et al., 2018). However, only a small portion of patients achieve survival benefits from immunotherapy. Therefore, a model to accurately predict the efficacy of immunotherapy and prognosis in GC patients is urgently needed.

Long noncoding RNA (lncRNA) is a type of RNA that is longer than 200 bp in length with no or little protein coding ability (Wang and Chang, 2011). LncRNA can influence gene expression at the epigenetic, transcriptional, and post-transcriptional levels. The dysregulation of lncRNA expression plays a crucial role in human carcinogenesis, and is expected to become a molecular target for tumor therapy and a biomarker for monitoring patient prognosis (Chandra Gupta and Nandan Tripathi, 2017; Zhang et al., 2019). Recent reports have demonstrated that lncRNAs are critical regulators of gene expression in the immune system (Chen et al., 2017) and involved in cancer progression by regulating the antitumor immune response (Hu et al., 2019; Xu et al., 2019). For example, in GC, linc-POU3F3 can activate TGF-β signal pathway, increase the ratio of T-reg *in vitro*, and promote the proliferation of GC cells (Xiong et al., 2015). Knock out lncRNA UCA1 could reduce PDL1 expression, increase IFNγ release and alleviate the immune suppressive effect in GC cells (Wang et al., 2019). Linc00936 expression was positively correlated with CD3+ and CD4+, and negatively correlated with CD8+ in peripheral blood of patients with GC. Linc00936/miR-425-5p/ZC3H12A axis functioned to promote cytokine induced killer cell cytotoxicity and suppress immune escape by decreasing the contents of immunosuppressive factors VEGF, IL-10, and TGF-β1 in GC (Li et al., 2021). Moreover, immune-related lncRNA (IRlncRNA) can be used to establish a model to predict prognosis in cancers (Chen et al., 2020; Luo et al., 2021; Wang et al., 2021; Zhao K. et al., 2021). However, due to difference in data processing, the absolute expression level of genes and lncRNAs between different datasets cannot be compared directly, and appropriate normalizations and standardizations of the lncRNA expression levels are required. Fortunately, researchers have found new methods to overcome these difficulties in processing data generated from different platforms. For example, the expression level of lncRNAs could be standardized and scaled by their relative rankings. One example of the application of these new methods, IRlncRNAPs has produced reliable results (Hong et al., 2020). Herein, we integrated an IRlncRNAPs prognostic model to predict the prognosis, tumor immune infiltration, and chemotherapeutic efficacy in GC.

## Materials and Methods

### Acquisition and Processing of Data and Clinical Information

The RNA-seq data (fragments per kilo base per million mapped reads [FPKM]) and the corresponding clinical follow-up information of GC were collected from TCGA database^1^, including 375 GC tissues and 32 normal adjacent tissues (Grossman et al., 2016). Ensembl gene IDs were converted into gene symbols, and the mRNAs and lncRNAs were distinguished by the GTF files which were acquired from Ensembl^2^ (Howe et al., 2021). A list of immune-related genes (IRGs) was downloaded from the ImmPort (Bhattacharya et al., 2018), and a co-expression strategy was used to screen IRlncRNAs.

### Determination of Differentially Expressed Immune-Related Long Noncoding RNAs

We performed our analysis using the *R* package *limma* and *pheatmap*, and IRlncRNAs with ∣Log2 fold change [FC]∣>2 and false discovery rate (FDR) <0.05 were defined as significantly differentially expressed immune-related long noncoding RNAs (DEIRlncRNAs).

### Construction and Validation of the Immune-Related Long Noncoding RNA Pairs-Based Prognostic Model

Immune-related long noncoding RNA pairs were identified from the DEIRlncRNAs as previously reported (Li et al., 2017). Specifically, pairwise comparisons were used to evaluate the lncRNA expression level in a specific sample and to generate a score for each IRlncRNAP. For each IRlncRNAP, a score of one indicated that the expression level of the former lncRNA was higher than the latter, otherwise the score was defined as zero. When the IRlncRNAPs were assigned the same score in more than 80% of the samples, these IRlncRNAPs were eliminated (Xiong et al., 2020). Next, the clinical information was extracted by removing data with a follow-up time of 0 days and combined with the relevant data of IRlncRNAPs using the *limma* package.

Univariate Cox analysis was used on the identified IRlncRNAPs to identify possible OS-related IRlncRNAPs by the *survival* package *R* 4.02, and IRlncRNAP with *P* < 0.01 was filtered for further analyses. Then, LASSO regression was performed using the *glmnet* package (Engebretsen and Bohlin, 2019) and multivariate Cox analysis was used to construct a prognostic model. Next, the riskScore of each sample was computed using the following formula:


$$R\,i\,s\,k\,S\,c\,o\,r\,e=\sum_{\text{i}=1}^{\text{k}}\beta_{\text{i}}\,{\text{S}}_{\text{i}}$$

where, *i* is the number of prognostic IRlncRNAPs, and βi and *Si* represent regression coefficients and IRlncRNAPs expression values, respectively.

The specificity and sensitivity of the prognostic model was assessed by the time-dependent ROC curve, and AUC for each ROC was calculated using the *survival ROC* package (Heagerty et al., 2000). The Akaike information criterion (AIC) values for each point of the 1-year ROC curve was calculated to identify the maximum inflection point, which was defined as the cut-off point of riskScores to divide GC patients into high- and low-risk groups. The log-rank test was performed to estimate OS differences between the two groups, and the Kaplan–Meier survival curve was drawn with *survival* and *survminer R* packages. Moreover, univariate and multivariate Cox regression analyses between the riskScore and clinicopathological characteristics (including age, gender, grade, and stage) was performed using the *survival* package to assess whether the riskScore could be used as an independent clinical prognostic factor, and the results are demonstrated as a forest map.

### Correlation of Clinical Features and RiskScore

To better understand the impact of the immune-related riskScore on tumor development and progression, subgroup analysis was applied to evaluate the correlations of the riskScore with clinical characteristics.

### Immune Features of Immune-Related Long Noncoding RNA Pairs

Immune infiltrate estimation data for TCGA tumors were obtained from the Tumor Immune Estimation Resource (TIMER) database including TIMER (Li T. et al., 2020), CIBERSORT (Newman et al., 2015), xCell (Aran et al., 2017), quanTIseq (Finotello et al., 2019), MCP-counter (Becht et al., 2016), and EPIC (Racle et al., 2017). The correlations of the riskScore and immune cells were calculated using Spearman’s correlation analysis and the Wilcoxon test. The results were presented in terms of a lollipop diagram and box charts. The analysis was performed using *ggplot2* package.

### Correlation of RiskScore and Expression of Tumor Immune-Related Genes

The *Ggstatsplot* package was used to explore the relationship between the riskScore and the expression of tumor IRGs and included other genes of interest. The results were illustrated using violin plots.

### Relationship Between the RiskScore and Gastric Cancer Treatment

To assess the significance of riskScore on guiding GC treatment, the half maximal inhibitory concentration (IC50) of chemotherapeutic drugs commonly used in GC treatment were calculated in high- and low-risk groups, respectively. Box charts obtained by *pRRophetic* (Geeleher et al., 2014) and *ggplot2* packages were used to show the results.

### Statistical Analysis

All statistical analyses were performed and graphics were drawn by using *R* software (version 4.0.2). Univariate and multivariate analysis were performed using the Cox regression model. The log-rank test was used for assessing the survival difference. For continuous variables, data comparisons among groups were made using Student’s *t*-test or the Wilcoxon rank sum test. Spearman’s correlation analysis and Wilcoxon signed-rank test was used to assess the relationships between immune cells and risk groups. Wilcoxon signed-rank test was adopted to compare the IC50 values of chemotherapeutic drugs between the high- and low- risk groups. For all statistical tests, *P-*values <0.05 were considered statistically significant.

## Results

### Identification of Differentially Expressed Immune-Related Long Noncoding RNAs in Gastric Cancer

**FIGURE 1:**
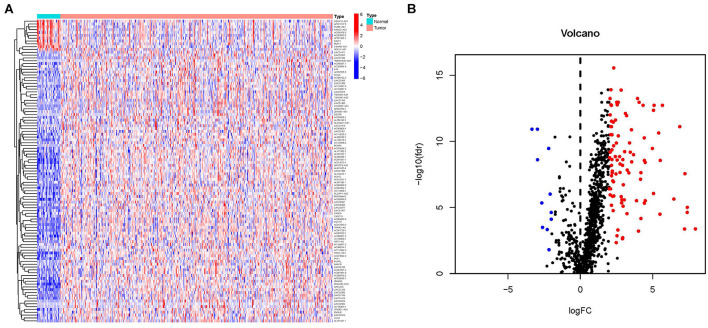
Differentially expressed immune-related long noncoding RNAs (DEIRlncRNAs) for GC. **(A)** Heatmap and **(B)** volcano plot showing upregulated (red) and downregulated (blue) DEIRlncRNAs between GC tissues and paracancerous tissues.

The transcriptome data of 375 GC tumor tissues and 32 para-cancerous tissues, together with their corresponding clinical information were downloaded from TCGA database. A total of 2483 IRGs were obtained from ImmPort, and their expression levels were extracted from TCGA transcriptome data. A co-expression strategy was used to obtain expression levels of IRlncRNAs. Correlation analysis was performed between all lncRNAs and IRGs. IRlncRNAs were selected if the correlation coefficient was more than 0.4 or less than −0.4 and the *P*-value was <0.001. We obtained the expression levels of 1082 IRlncRNAs. Among these, 107 IRlncRNAs were differentially expressed in GC and paracancerous tissues (FDR <0.05 and ∣ log2 FC ∣>2) (Figure 1A), of which 96 were up-regulated and 11 were down regulated (Figure 1B and Supplementary Table 1).

### Identification of Overall Survival-Related Immune-Related Long Noncoding RNA Pairs and Construction of the Prognostic Model

**FIGURE 2:**
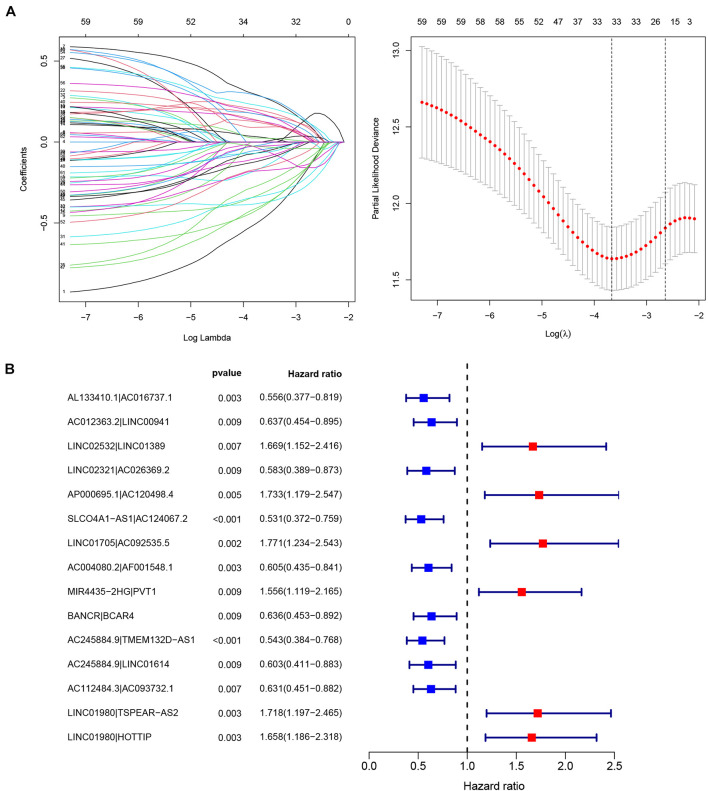
Construction of the prognostic model. **(A)** Predictor selection by the LASSO and **(B)** forest plots of the 15 OS-related immune-related long noncoding RNA pairs (IRlncRNAPs) identified by Cox proportional hazard regression, the represented prognostic IRlncRNAPs with hazard ratios >1 are shown as red dots and the represented prognostic IRlncRNAPs with hazard ratios <1 are shown as blue dots.

By recombining the 107 DEIRlncRNAs, 4297 IRlncRNAPs and their relative expression levels were obtained. The *limma* package was used to filter out patients with zero survival time and with incomplete clinical data. These data were merged with IRlncRNAPs to obtain survival information and IRlncRNAPs expression of 350 patients. In total, 62 OS-related IRlncRNAPs in GC patients were identified by univariate Cox analysis (*P* < 0.01) (Supplementary Table 2), while 33 OS-related IRlncRNAPs were extracted after 1,000 iterations using LASSO Cox regression analysis (Figure 2A). Finally, we constructed an IRlncRNAP risk prognostic signature consisting of 15 OS-related IRlncRNAPs (AL133410.1∣ AC016737.1, AC012363.2∣LINC00941, LINC02532∣LINC01389, LINC02321∣AC026369.2, AP000695.1∣AC120498.4, SLCO4A1-AS1∣AC124067.2, LINC01705∣AC092535.5, AC004080.2∣AF0 01548.1, MIR4435-2HG∣PVT1, BANCR∣BCAR4, AC245884.9∣ TMEM132D-AS1, AC245884.9∣LINC01614, AC112484.3∣AC 093732.1, LINC01980∣TSPEAR-AS2, and LINC01980∣HOTTIP) using a Cox proportional hazards model (Figure 2B) and the coefficients of each IRlncRNAP were obtained from the model (Table 1).

**TABLE 1:** Prognostic IRlncRNAPs and their corresponding coefficients.

ID	Coefficient	ID	Coefficient
AL133410.1∣AC016737.1	–0.73841	MIR4435-2HG∣PVT1	0.336364
AC012363.2∣LINC00941	–0.3917	BANCR∣BCAR4	–0.59577
LINC02532∣LINC01389	0.563555	AC245884.9∣TMEM132D-AS1	–0.43574
LINC02321∣AC026369.2	–0.49454	AC245884.9∣LINC01614	–0.3349
AP000695.1∣AC120498.4	0.473551	AC112484.3∣AC093732.1	–0.5939
SLCO4A1-AS1∣AC124067.2	–0.4365	LINC01980∣TSPEAR-AS2	0.425801
LINC01705∣AC092535.5	0.448092	LINC01980∣HOTTIP	0.449463
AC004080.2∣AF001548.1	–0.63962		

### Validation of the Immune-Related Long Noncoding RNA Pair Signature

**FIGURE 3:**
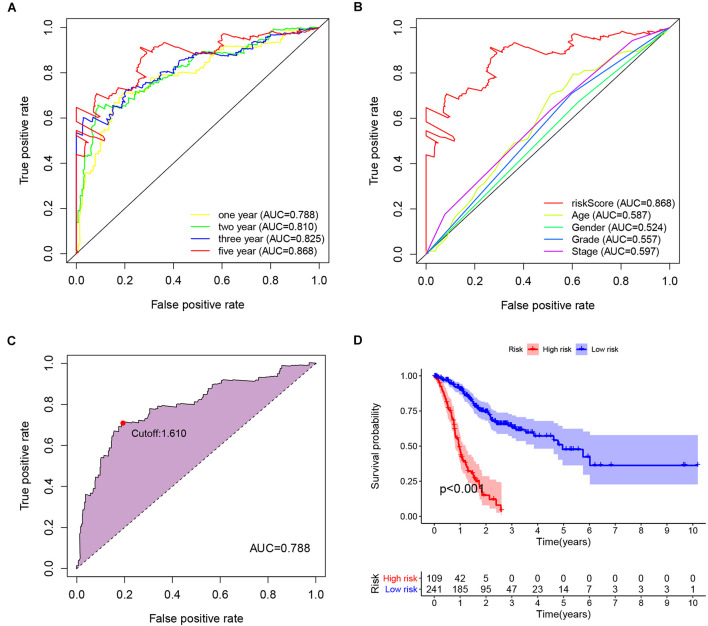
Prognostic value of the IRlncRNAPs signature. **(A)** Time-dependent ROC curve analysis of riskScores and **(B)** the respective 5-year ROC curves of riskScores and other common clinical characteristics. **(C)** The cut-off point of the riskScore on the 1-year ROC curve was 1.610. **(D)** Kaplan–Meier survival curve analysis for the high- and low-risk group based on the riskScore.

A riskScore for each patient was computed (Supplementary Table 3). We used the riskScore to draw the ROC curves of patients and to calculate the respective AUC values. The AUC values for the 1-, 2-, 3-, and 5-year ROC curve of the riskScores were 0.788, 0.810, 0.825, and 0.868, respectively (Figure 3A), which were greater than 0.75, indicating a high specificity and sensitivity of this prognostic signature. In addition, compared with clinical features such as age, gender, grade, and TNM stage, our IRlncRNAPs signature generated the largest AUC value (Figure 3B).

**FIGURE 4:**
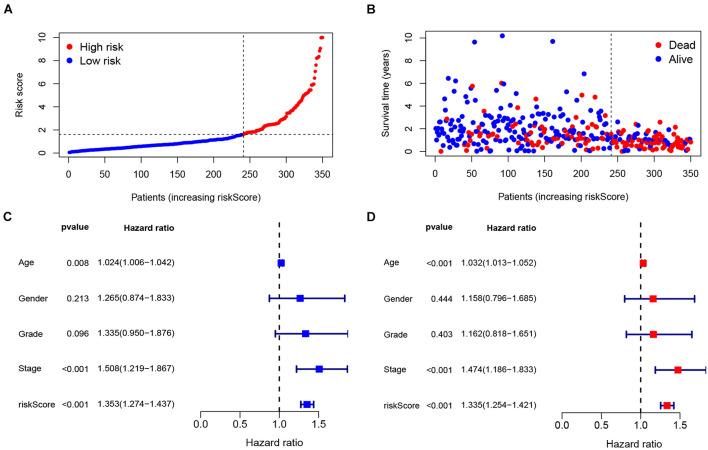
Validation of the IRlncRNAPs signature. **(A)** RiskScore distribution of GC patients. **(B)** Survival status of GC patients with increasing riskScore. **(C)** Univariate and **(D)** multivariate Cox regression analyses of the riskScore.

The maximum inflection point in the 1-year ROC curve was recognized (Figure 3C), and was set as the cut-off point. Base on the optimal cut-off value 1.610 of the riskScore, 109 GC patients were assigned to the high-risk group, and the remaining 241 cases were assigned to low-risk group. Survival curve was drawn to evaluate the survival of the patients, and the data indicated that prognosis of the high-risk group was poorer than that of the low-risk group (*P* < 0.001, Figure 3D). The riskScore and survival outcomes of each case were shown in Figures 4A,B. Patients in the high-risk group showed shorter survival time, and most of the deaths were distributed in the high-risk group (Figures 4A,B). We evaluated the prognostic value of clinicopathological characteristics (age, gender, grade, and TNM stage) and IRlncRNAPs riskScore by univariate Cox regression analysis, the results illustrated a strong association between the riskScore and the OS of GC patients (HR = 1.353, 95%CI 1.274–1.437, and *P* < 0.001) (Figure 4C). Furthermore, the multivariate regression Cox analysis (Figure 4D) suggested that the riskScore remained as an independent prognostic factor after adjusting for age, gender, tumor stage, and tumor grade (HR = 1.335, 95%CI 1.254–1.421, and *P* < 0.001). The above datas indicated that our IRlncRNAPs prognosis signature had good predictive value for prognosis in GC.

### Relationship Between the Immune-Related Long Noncoding RNA Pairs Signature and Clinical Characteristics

**FIGURE 5:**
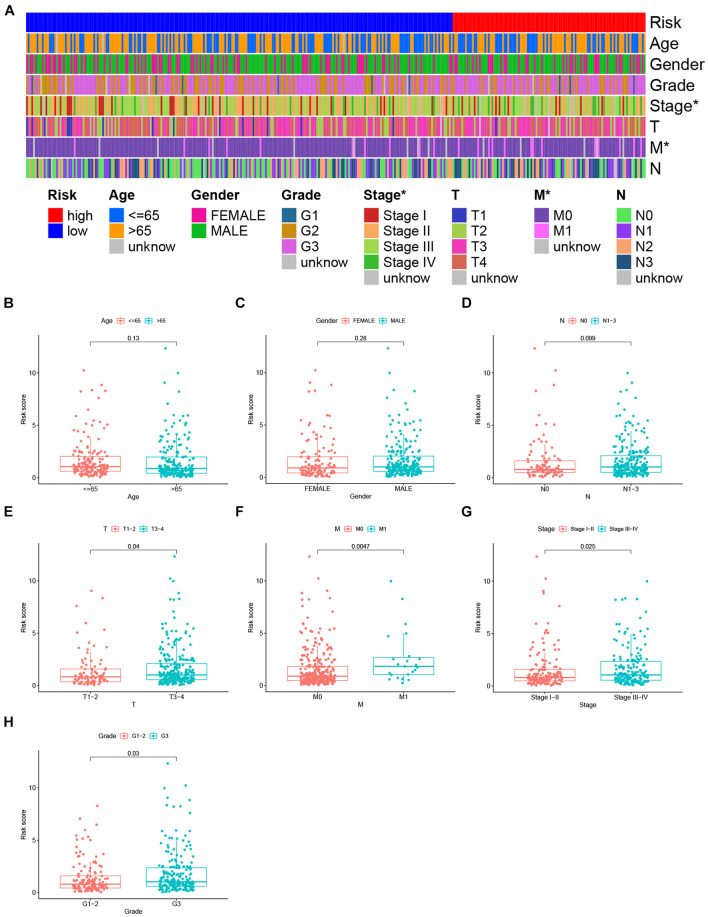
Correlation of the clinical features and IRlncRNAPs signature. **(A)** Heat map showing the distribution of clinical features between the high- and low-risk groups. The riskScores in different **(B)** age, **(C)** gender, **(D)** N stage, **(E)** T stage, **(F)** M stage, **(G)** clinical stage, and **(H)** tumor grade groups for GC patients. *P < 0.05.

Next, the correlation between the riskScore of GC patients and clinical characteristics were computed. The heat map showed the distributions of age, gender, and tumor grade and stage (TMN) in the two groups (Figure 5A), and significant differences were found in tumor stage (*P* < 0.05) and M stage (*P* < 0.05). Subsequently, as shown in the box plot, the patient’s age (Figure 5B), gender (Figure 5C), and lymph node metastasis (N stage) (Figure 5D) were not significantly correlated with riskScore, but the depth of tumor invasion (T stage) (Figure 5E), metastasis (M stage) (Figure 5F), tumor stage (Figure 5G), and degree of tumor differentiation (Grade) (Figure 5H) were significantly correlated with the riskScore. Patients with higher pathological stage (T3-4), distant metastasis (M1 stage), advanced-stage (Stage III–IV) and poor differentiation (Grade 3) had higher riskScores. These clinical features showed a good correlation with our IRlncRNAPs signature, and the riskScore was often higher when the tumors had a high degree of malignancy.

### Immune Features of Immune-Related Long Noncoding RNA Pairs Signature in Gastric Cancer

**FIGURE 6:**
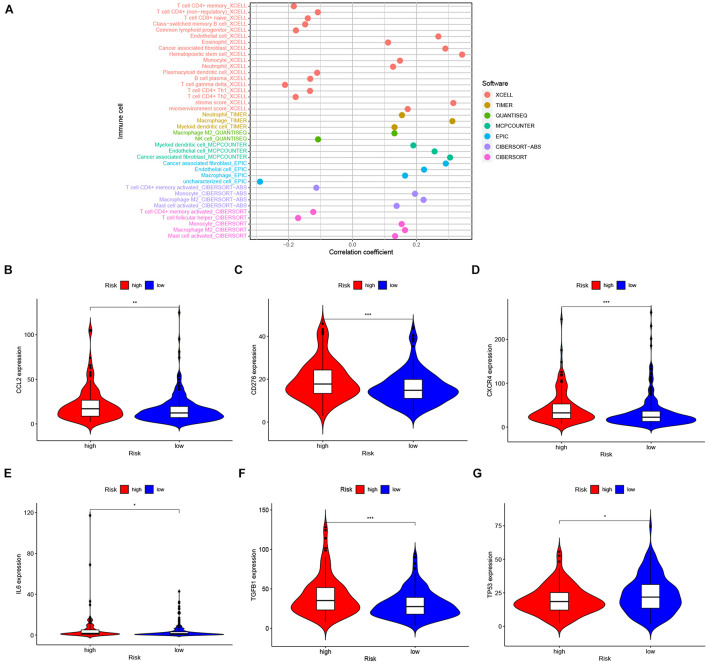
Immune features of IRlncRNAPs signature. **(A)** Correlation between the riskScore and tumor-infiltrating immune cells, as shown in a lollipop diagram. Expression patterns of the IRGs between high- and low-risk groups, the expression of **(B)** *CCL2*, **(C)** *CD276*, **(D)** *CXCR4*, **(E)** *IL6*, and **(F)** *TGFβ1* increased in the high-risk group and **(G)** *TP53* decreased in high-risk group. *P < 0.05, **P < 0.01, ***P < 0.001, and ns, no significance.

Our signature was constructed on the basis of the IRlncRNA, thus we also explored the correlation of the IRlncRNAPs signature and the tumor immune microenvironment (TIME), and whether our model could predict the profile of immune cell infiltration in GC. The bubble plot (Figure 6A) demonstrated that the riskScore was positively correlated with tumor associated fibroblasts (CAFs), monocytes, M2 macrophages, myeloid dendritic cells, endothelial cells and other immune cells, and showed a negative correlation with CD4^+^ T cells, Natural Killer (NK) cells, and plasma B cells (Supplementary Table 4). Furthermore, the box plot (Supplementary Figure 1) revealed that the level of immune cells differed significantly between the two groups of patients.

Since immunotherapy has achieved promising effects in the treatment of solid tumors including GC (Kang et al., 2017; Fuchs et al., 2018), we extracted the expression data of immunotherapy-related genes as well as other cancer driver genes. As shown in the violin chart, when compared with the low-risk group, the expression of *CCL2*, *CD276*, *CXCR4*, *IL6*, and *TGFβ1* were significantly upregulated (Figures 6B–F), while the expression of *TP53* was downregulated significantly in the high-risk group (Figure 6G). The expressions of *PDCD1*, *CD274*, *PDCD1LG2*, and *CTLA4* were not significantly different between the two groups (Supplementary Figure 2).

### Relationship Between Immune-Related Long Noncoding RNA Pairs Signature and Chemosensitivity

**FIGURE 7:**
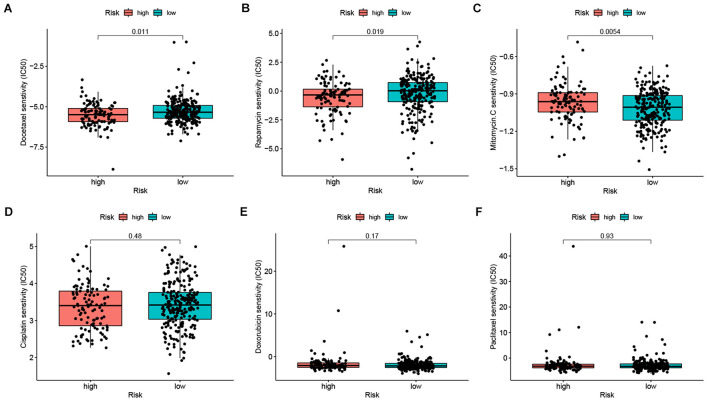
The chemosensitivity of high- and low-risk groups. The high-risk group was related to a lower IC50 for **(A)** docetaxel, **(B)** rapamycin and high IC50 for **(C)** mitomycin C, whereas the IC50 of **(D)** cisplatin, **(E)** doxorubicin, and **(F)** paclitaxel showed no significant difference between the two groups.

Chemotherapy is an important treatment for GC. Thus, we investigated whether our IRlncRNAPs signature could predict the sensitivity of patients to chemotherapy drugs, in order to better guide clinical practice. The IC50 of chemotherapy drugs commonly used in GC between the two groups were computed and compared. In the high-risk group, the IC50 of docetaxel (Figure 7A) and rapamycin (Figure 7B) was lower, while that of mitomycin C (Figure 7C) was higher. There was no significant difference in the sensitivity of cisplatin (Figure 7D), doxorubicin (Figure 7E), or paclitaxel (Figure 7F) between the two groups. This result suggested that patients with a higher riskScore were more sensitive to docetaxel and rapamycin, and may benefit more from chemotherapy with these two agents.

## Discussion

Gastric cancer has a high incidence and mortality rates in East Asia (Yang et al., 2018; Sung et al., 2021). At present, many prognostic models have been established to predict the prognosis of GC patients. Prognostic models constructed on the basis of absolute gene expression may not be suitable for direct comparison due to differences in sequencing methods that have generated different data sets, and thus weakens their predictive value. Prognostic models constructed on relative rankings of gene transcript expression level may overcome these differences across data groups caused by different sequencing platforms, and can be used to compare different data sets (Li et al., 2017; Zhao E. et al., 2021).

In recent years, researchers have gradually realized that the heterogeneity of tumors is the primary reason for drug resistance and treatment failure (Burrell et al., 2013). In addition to the heterogeneity of tumor cells, the complex tumor microenvironment (TME) also has a significant effect on the occurrence, progression, and development of drug resistance of tumors (Junttila and de Sauvage, 2013; Quail and Joyce, 2013). The TIME is an important element characterizing the TME and has a profound effect on tumor progression (Hinshaw and Shevde, 2019), in addition, cancer immunotherapy has brought new hope to patients with advanced cancers (Iwai et al., 2005; Sharma and Allison, 2015). A precise evaluation of the tumor is the premise for precise treatment. Combined positive score (CPS) has been applied to the immunotherapy of GC (Kulangara et al., 2019). More accurate immune-related models and scores are urgently needed to evaluate prognosis, immune cell infiltration, and therapeutic effects of GC patients. LncRNAs are well known players in tumor progression (Huarte, 2015), furthermore, lncRNAs are also involved in the anti-tumor immune response, infiltration of immune cells, and in tumor immunosuppression (Luo et al., 2020). Therefore, IRlncRNAs have increasingly been adopted as biomarkers to diagnose tumors and to predict their prognosis, and it is believed that IRlncRNAs could be targets for tumor immunotherapy (Li Y. et al., 2020; Wang et al., 2021; Zhao K. et al., 2021). Thus, we established a prognostic model based on IRlncRNAPs in GC, and explored its predictive ability for survival of GC patients, and evaluated the clinical characteristics, immune cells infiltration, and chemosensitivity of GC patients based on our immune riskScore.

Herein, we obtained the transcriptomic and clinical characteristics of GC from TCGA, and collected IRGs according to the ImmPort database. In GC RNA-seq data, we selected lncRNAs that were significantly co-expressed with IRGs as IRlncRNAs, and obtained 107 DEIRlncRNAs. Next, 4,297 IRlncRNAPs were identified. After univariate/multivariate Cox and LASSO regression analysis of these 4,297 IRlncRNAPs, we obtained a prognostic model consisting of 15 OS-related IRlncRNAPs in GC. We calculated the riskScore for each patient based on the prognostic model and performed ROC curve analysis. The area of the 1-, 2-, 3-, and 5-year ROC curves of the respective riskScores were 0.788, 0.810, 0.825, and 0.868, suggesting that the model had a strong predictive ability for prognosis of GC patients. With the extension of the follow-up time, the predictive power of this new model was stronger, and the predictive efficiency of IRlncRNAPs was better than other clinicopathological characteristics such as age, gender, tumor stage, and degree of tumor differentiation.

Multivariate Cox regression analysis also showed that the IRlncRNAPs signature functioned as an independent risk factor for GC. According to the riskScore, patients could be stratified into high- and low-risk groups. Kaplan–Meier survival analysis found that the high-risk group had significantly poorer OS, and for tumors with poorer differentiation and higher malignancy (T3-4 stage, M1 stage, Grade 3, and Stage III–IV), the riskScores was higher. The above results indicated that our model had a good predictive power for the prognosis of GC patients, and had a good discrimination for malignant degree of the tumors.

Tumor infiltrating immune cells have an impact on cancer development, and influence the response of immunotherapy and prognosis of patients; therefore, the analysis of tumor infiltrating cells may reveal the mechanisms of immune escape adopted by tumor cells and may provide new targets for immunotherapy (Fridman et al., 2017; Wouters and Nelson, 2018; Zhang and Zhang, 2020). We explored the correlation between riskScore and immune cells infiltration. We found that the riskScore showed a positive correlation with CAFs, monocytes, M2 macrophages, myeloid dendritic cells, and endothelial cells, and showed a negative correlation with the infiltration of CD4^+^ T cells, NK cells, and plasma B cells. CAFs represent the key cellular components of the tumor stroma, and participate in the progression of a variety tumors, including GC (Yan et al., 2015; Chen and Song, 2019; Ham et al., 2019). Furthermore, CAFs also participate in the regulation of lncRNA, leading to the poor prognosis of tumor patients (Zhao et al., 2017). Monocyte recruitment to tumors are differentiated into tumor-associated macrophages such as M2 macrophages to promote cancer progression (Qian et al., 2011; Arwert et al., 2018; Larionova et al., 2020). Cancer cells can also recruit and induce myeloid dendritic cells to differentiate into a regulatory dendritic cell subset, which constitutes the tumor immunosuppressive microenvironment and helps tumors escape from immune control (Liu et al., 2009). Endothelial cells contribute to the formation of new blood vessels which supply nutrients for tumor cells and provide gateway for tumor metastasis and, thus contribute to tumor progression (Hida et al., 2018). Our findings are consistent with these previously reported results. The higher the riskScore of patients, the greater the infiltration of pro-tumor immune cells, and the worse outcome of patients. In addition, CD4^+^ T cells are also essential to the anti-tumor immune response (Pardoll and Topalian, 1998; Velders et al., 2003), which can directly recognize peptide/class II MHC on the surface of cancer cells and kill them (Echchakir et al., 2000). NK cells are important for tumor immune surveillance, and the occurrence of malignant tumors has been associated with primary NK cell immunodeficiency (Lorenzi et al., 2013). Plasma cells in the TME can release a variety of antibodies, and induce phagocytosis and antibody-dependent cellular cytotoxicity (ADCC) to promote anti-tumor immunity (Kurai et al., 2007; Gilbert et al., 2011). Herein, these immune cells were inversely associated with the patient’s riskScore. In patients with a higher riskScore, the infiltration of anti-tumor immune cells, widely believed to promote tumor immune response and inhibit tumor progression, was lower than in the low riskScore group, which may be the cause of a poorer outcomes observed in these patients. Our prognosis model was closely related to tumor immunity, and well correlated with the infiltration status of immune cells and the prognosis of GC patients. Patients in the high-risk group exhibited a higher level of pro-tumor immune cells infiltration and a lower level of anti-tumor immune cells infiltration, which indicated that the tumor was in an immunosuppressive state, conducive to tumor progression. The latter may explain why the high-risk group of GC patients had a poor prognosis based on this new model.

We then examined the relationship between the riskScore and IRGs’ expression. In the high-risk group, patients showed significantly higher expression of *CCL2*, *CD276*, *CXCR4*, *IL6* and *TGFβ1*, and a lower expression of *TP53*. Chen et al. (2018) reported that lncRNA-LNMAT1 could upregulate *CCL2* expression, which resulted in enhancement of macrophages recruitment and promoted the phenotypic switch of macrophages toward a tumor-associated macrophage phenotype facilitating lymph node metastasis of cancer cells, in lymph node positive bladder cancer. CD276 is an immune checkpoint that can inhibit the function of T cells, facilitates tumor immunity escape, and promotes cancer progression (Chen et al., 2013; Dai et al., 2014; Guo et al., 2019). CXCR4 is a well-known chemokine receptor, expressed by lymphocytes, hematopoietic stem cells, endothelial cells, epithelial cells, and cancer cells, and is associated with tumor progression, angiogenesis, metastasis, and poor survival (Teicher and Fricker, 2010). Further, CXCR4 is also important for the progression of GC and is associated with poor outcome in GC (Xiang et al., 2017). IL6 is a cytokine present in the TME. The expression of *IL6* is up-regulated in many tumors, and contributes to facilitate tumor growth and is related to tumor drug chemoresistance (Kumari et al., 2016). In many tumors, the dysregulated expression of *TGFβ1* has led to changes in the number and function of tumor-infiltrated immune cells, and promotion of tumor immune escape mechanisms (Hargadon, 2016). *TP53* is a well-known cancer suppressor gene, which can inhibit *CXCR4* expression and reduces cancer cell migration (Mehta et al., 2007). In mouse pancreatic cancer models, the loss of p53 function promotes STAT3 phosphorylation by increasing the secretion of IL6, which regulates immune cell infiltration in the TME, promotes tumor progression and immune escape (Wormann et al., 2016). These studies were consistent with our findings, whereby the expression of *TP53* is decreased in the high-risk group, while the expressions of *CCL2*, *CD276*, *CXCR4*, *IL6*, and *TGFβ1* are increased, which indicates these genes may interact with each other, altering the TIME and distribution of immune infiltrating cells, promoting tumor immune escape and tumor progression in GC. Therefore, our IRlncRNAPs signature has a good predictive value for IRGs expression and immune microenvironment of GC. We believe that our model will provide significant value to improve the efficacy of immunotherapy.

Finally, we investigated the relationship between the IRlncRNAPs signature and chemotherapy sensitivity of GC, and found that docetaxel and rapamycin exhibited a lower IC50 in the high-risk group, while mitomycin C had a higher IC50. The IC50 of cisplatin, doxorubicin, and paclitaxel showed no significant differences across the two groups. Therefore, our IRlncRNAPs signature also possesses a certain predictive value for the chemotherapy sensitivity of GC patients.

Of course, our model also has some limitations that should be taken into consideration. First, the reliability of the model requires further validation by external data. Our model was constructed based on information extracted from TCGA database. In other commonly used tumor databases, there is insufficient lncRNA data in Gene Expression Omnibus (GEO) database to allow validation studies, and the International Cancer Genome Consortium (ICGC) database lacks transcriptome data for GC. Therefore, there is no additional dataset suitable for external validation at present. However, it should be noted that we used the ratio of lncRNA pairs in the samples to construct the model, and applied a variety of methods to repeatedly validate its effectiveness. Thus we believe that our model is reliable. Of course, we hope to use GC samples from our own patients in an independent large-scale sequencing study and clinical follow-up in the future to confirm the reliability of this new model, but understandably this will take time. Secondly, the functions and specific mechanisms of these lncRNAs still need to be experimentally verified *in vitro* and *in vivo*. Finally, our study is still a retrospective study, and additional cases need to be included, while prospective clinical studies should also be foreseen.

## Conclusion

We identified an effective and practical prognostic riskScore model based on 15 IRlncRNAPs in GC. This model showed good performance in predicting outcome of GC patients and in evaluating immune cells infiltration and chemotherapy sensitivity in GC. We hope that this model can be applied to the clinic in future, in order to effectively guide immuno- and chemo-therapy management, and predict the survival of GC patients.

## Data Availability Statement

The datasets presented in this study can be found in online repositories. The names of the repository/repositories and accession number(s) can be found in the article/Supplementary Material.

## Author Contributions

SS, SL, and ZW performed the bioinformatics analysis. CZ, BL, and YH conceptualized and designed the study. DM and XJ contributed to the analysis tools. SS, SL, and BL wrote the first draft of the manuscript. All authors wrote sections of the manuscript, and contributed to the article and approved the submitted version.

## Conflict of Interest

The authors declare that the research was conducted in the absence of any commercial or financial relationships that could be construed as a potential conflict of interest.

## Publisher’s Note

All claims expressed in this article are solely those of the authors and do not necessarily represent those of their affiliated organizations, or those of the publisher, the editors and the reviewers. Any product that may be evaluated in this article, or claim that may be made by its manufacturer, is not guaranteed or endorsed by the publisher.

## Footnotes

1
https://portal.gdc.cancer.gov/

2
http://asia.ensembl.org

## Supplementary Material

Supplementary Figure 1 Differential distribution of immune infiltrating cells in high- and low-risk group (*P* < 0.05).

Supplementary Figure 2 There were no difference in the expressions of these genes between the high- and low-risk group (ns, no significance).

Supplementary Table 1 DEIRlncRNAs in GC and paracancerous tissues (FDR <0.05 and ∣ log2 FC ∣ >2).

Supplementary Table 2 62 OS-related IRlncRNAPs in GC (*P* < 0.01).

Supplementary Table 3 The riskScore of each GC patient.

Supplementary Table 4 Correlation coefficients between riskScore and immune infiltrating cells (*P* < 0.05).
