# Supplementary figures and images for: Identification of an Immune-Related Long Noncoding RNA Pairs Model to Predict Survival and Immune Features in Gastric Cancer
Source: Front Cell Dev Biol. 2021 Sep 21;9:726716. doi: 10.3389/fcell.2021.726716 (PMC8491937; doi:10.3389/fcell.2021.726716)

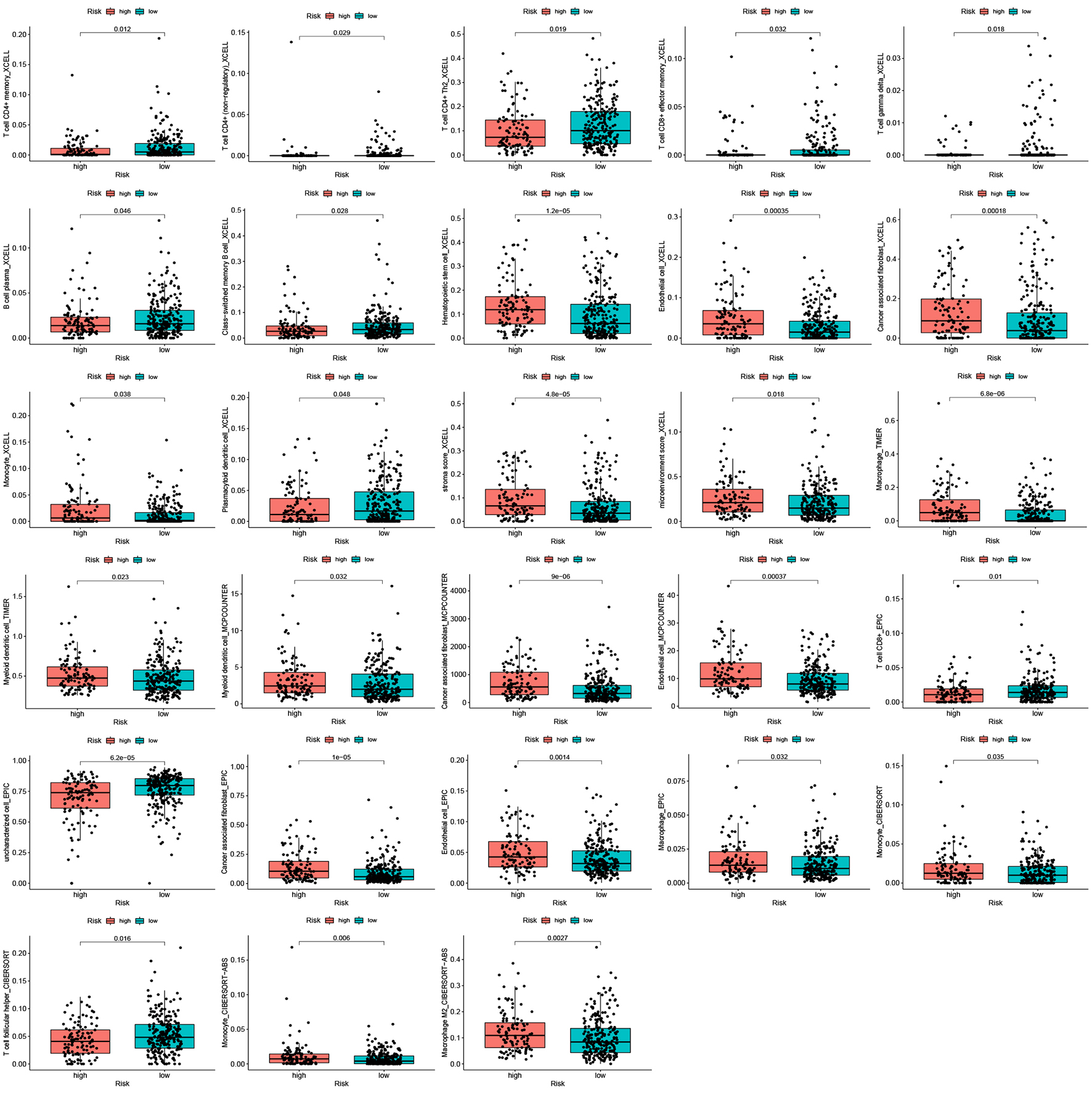

Supplement: Supplementary Figure 1 — Differential distribution of immune infiltrating cells in high- and low-risk group (P < 0.05). [file Image_1.JPEG]

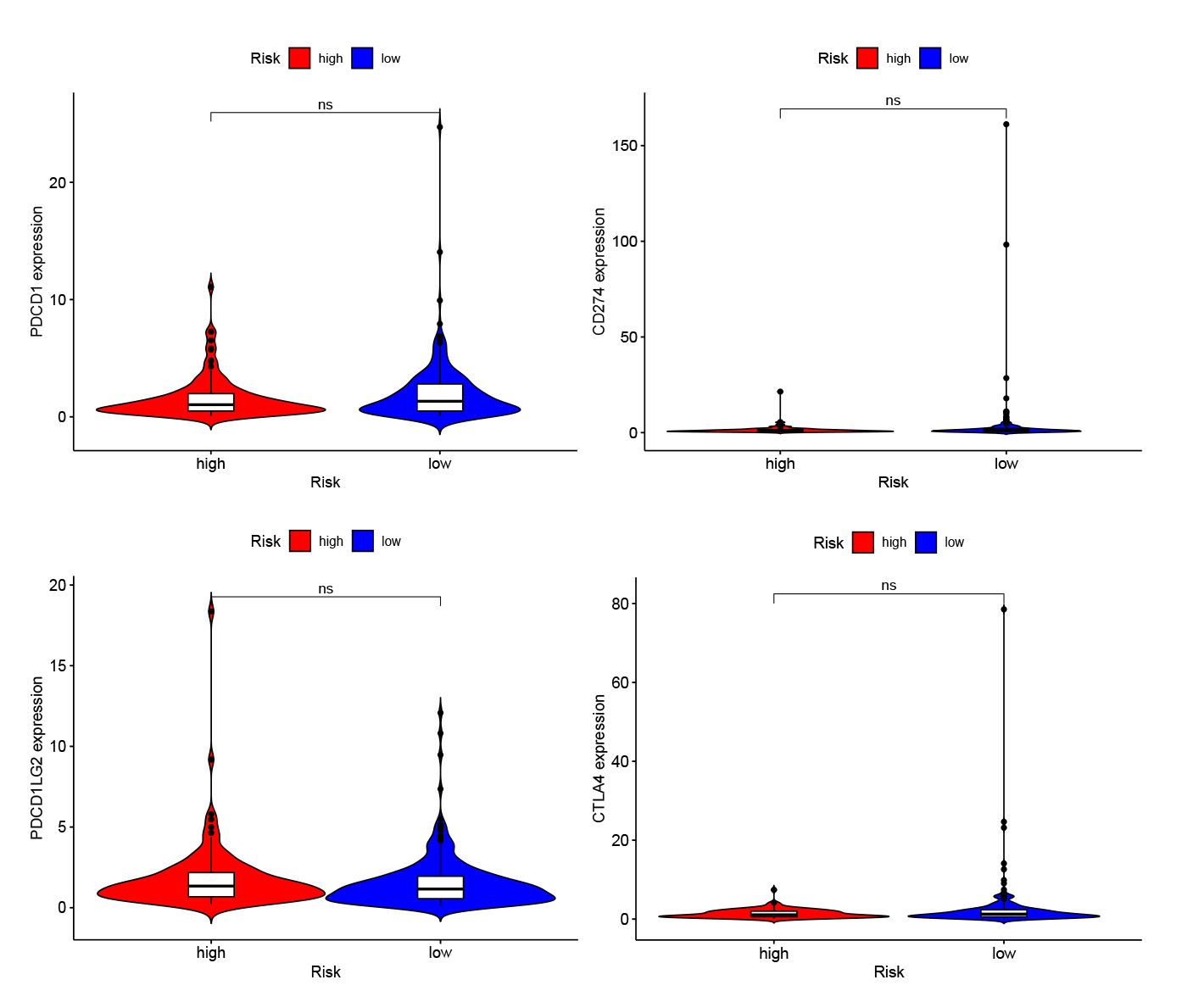

Supplement: Supplementary Figure 2 — There were no difference in the expressions of these genes between the high- and low-risk group (ns, no significance). [file Image_2.JPEG]
