# Supplementary material for: Identification of an Immune-Related Long Noncoding RNA Pairs Model to Predict Survival and Immune Features in Gastric Cancer
Source: Front Cell Dev Biol. 2021 Sep 21;9:726716. doi: 10.3389/fcell.2021.726716 (PMC8491937; doi:10.3389/fcell.2021.726716)
Supplement: Supplementary Table 2 — 62 OS-related IRlncRNAPs in GC (P < 0.01). [file Table_2.DOCX]

| lncRNAP | HR | HR.95L | HR.95H | pvalue |
| --- | --- | --- | --- | --- |
| AL133410.1\|AC016737.1 | 0.555556647 | 0.376812 | 0.81909 | 0.003003 |
| AL133410.1\|LINC01705 | 0.629131766 | 0.452941 | 0.87386 | 0.005705 |
| AL133410.1\|AC036108.3 | 0.617020344 | 0.427381 | 0.890808 | 0.009964 |
| AL133410.1\|LINC01614 | 0.643683359 | 0.462546 | 0.895757 | 0.008978 |
| AC012363.2\|LINC00941 | 0.63715433 | 0.453688 | 0.894813 | 0.009285 |
| AC012363.2\|LINC01614 | 0.624686507 | 0.441109 | 0.884664 | 0.008043 |
| LINC02532\|LINC01389 | 1.668628876 | 1.152297 | 2.416323 | 0.00672 |
| LINC02532\|HOTTIP | 1.569766513 | 1.129851 | 2.180967 | 0.007196 |
| LINC02321\|AC026369.2 | 0.582900484 | 0.389045 | 0.873352 | 0.008886 |
| AC090192.2\|AC112484.3 | 1.630202484 | 1.154786 | 2.301345 | 0.005469 |
| AC012073.1\|AP000695.1 | 0.482366016 | 0.33203 | 0.700771 | 0.00013 |
| PGM5-AS1\|LINC01389 | 1.633533906 | 1.12921 | 2.363097 | 0.009187 |
| AP000695.1\|AL109615.3 | 1.567268402 | 1.126402 | 2.180686 | 0.00767 |
| AP000695.1\|HOXC-AS1 | 1.568448447 | 1.129176 | 2.178607 | 0.007262 |
| AP000695.1\|AC004080.2 | 1.740862733 | 1.246168 | 2.431938 | 0.001153 |
| AP000695.1\|AC120498.4 | 1.733071049 | 1.179018 | 2.547489 | 0.005144 |
| AP000695.1\|AC112484.3 | 1.661321278 | 1.175953 | 2.347022 | 0.003985 |
| AP000695.1\|AC079684.2 | 1.650092808 | 1.186332 | 2.295146 | 0.002931 |
| AP000695.1\|LINC01389 | 1.778994123 | 1.259584 | 2.512591 | 0.001075 |
| AP000695.1\|AL117382.2 | 1.580468568 | 1.115806 | 2.238634 | 0.00997 |
| AP000695.1\|AC091057.1 | 2.031407946 | 1.425079 | 2.895711 | 8.91E-05 |
| AP000695.1\|SCAT2 | 1.68723586 | 1.212717 | 2.347427 | 0.001905 |
| AP000695.1\|LINC01558 | 1.682319263 | 1.158332 | 2.443339 | 0.006297 |
| AL034550.1\|LINC01614 | 0.611921538 | 0.438671 | 0.853596 | 0.003827 |
| LINC01235\|AC004264.1 | 1.605012772 | 1.154391 | 2.231536 | 0.004896 |
| LINC01235\|LINC01389 | 1.614251878 | 1.156319 | 2.253539 | 0.004905 |
| LINC01235\|AC091057.1 | 1.699838701 | 1.220111 | 2.368187 | 0.001713 |
| AC068491.3\|LINC01705 | 0.646318799 | 0.465221 | 0.897913 | 0.009271 |
| HAND2-AS1\|LINC01389 | 1.670165455 | 1.143134 | 2.44018 | 0.008014 |
| HAND2-AS1\|TSPEAR-AS2 | 1.643805827 | 1.157624 | 2.334175 | 0.005468 |
| SLCO4A1-AS1\|AC124067.2 | 0.531253477 | 0.371855 | 0.758979 | 0.000511 |
| NALT1\|LINC01614 | 0.55986856 | 0.392607 | 0.798388 | 0.001358 |
| LINC01705\|LMNTD2-AS1 | 1.564954552 | 1.125996 | 2.175036 | 0.007664 |
| LINC01705\|AC092535.5 | 1.771292367 | 1.23379 | 2.542958 | 0.001944 |
| AC004080.2\|AF001548.1 | 0.604757597 | 0.434747 | 0.841253 | 0.002822 |
| AC004080.2\|NKILA | 0.621514288 | 0.437234 | 0.883463 | 0.008038 |
| AC004080.2\|AC092535.5 | 0.586730379 | 0.398937 | 0.862924 | 0.006748 |
| AC004080.2\|LINC00941 | 0.548945502 | 0.387655 | 0.777343 | 0.000727 |
| AC004080.2\|LINC01614 | 0.618378719 | 0.441258 | 0.866596 | 0.005246 |
| MIR4435-2HG\|PVT1 | 1.556408862 | 1.118994 | 2.164809 | 0.008593 |
| BANCR\|BCAR4 | 0.635755195 | 0.45298 | 0.892279 | 0.008819 |
| AC245884.9\|LINC01980 | 0.634836234 | 0.456203 | 0.883417 | 0.007034 |
| AC245884.9\|TMEM132D-AS1 | 0.542558375 | 0.383532 | 0.767522 | 0.00055 |
| AC245884.9\|LINC01614 | 0.602885168 | 0.411467 | 0.883354 | 0.009422 |
| AC120498.4\|LINC01614 | 0.598569773 | 0.408757 | 0.876524 | 0.00836 |
| AC112484.3\|LINC01980 | 0.61814469 | 0.436127 | 0.876128 | 0.00687 |
| AC112484.3\|AC093732.1 | 0.631057896 | 0.45139 | 0.88224 | 0.007084 |
| AC112484.3\|C5orf66-AS1 | 0.646572117 | 0.464624 | 0.899771 | 0.009699 |
| AC112484.3\|LINC00941 | 0.605744694 | 0.428776 | 0.855754 | 0.004461 |
| AC079684.2\|PART1 | 0.612593215 | 0.426711 | 0.879449 | 0.007901 |
| AC079684.2\|LINC01614 | 0.633484213 | 0.452653 | 0.886555 | 0.007765 |
| LINC01389\|AL356740.3 | 0.594583065 | 0.415428 | 0.850999 | 0.004484 |
| LINC01389\|LINC01614 | 0.622616597 | 0.448291 | 0.864732 | 0.004697 |
| LINC01980\|TSPEAR-AS2 | 1.717807473 | 1.197112 | 2.464985 | 0.003321 |
| LINC01980\|HOTTIP | 1.658106493 | 1.185892 | 2.318354 | 0.003107 |
| KCNMB2-AS1\|AL117382.2 | 1.657247597 | 1.157503 | 2.372754 | 0.005803 |
| HOTTIP\|AC124067.2 | 0.609519698 | 0.436284 | 0.851542 | 0.003709 |
| HOTTIP\|LINC01614 | 0.624963133 | 0.444438 | 0.878816 | 0.006878 |
| SMIM25\|LINC01614 | 0.561144673 | 0.392308 | 0.802643 | 0.001557 |
| AC114488.1\|LINC01614 | 0.630737035 | 0.451972 | 0.880208 | 0.006721 |
| AC091057.1\|AC026369.2 | 0.629936624 | 0.443284 | 0.895183 | 0.009951 |
| SCAT2\|LINC01614 | 0.593690876 | 0.42501 | 0.829319 | 0.002233 |
