# Supplementary material for: Identification of an Immune-Related Long Noncoding RNA Pairs Model to Predict Survival and Immune Features in Gastric Cancer
Source: Front Cell Dev Biol. 2021 Sep 21;9:726716. doi: 10.3389/fcell.2021.726716 (PMC8491937; doi:10.3389/fcell.2021.726716)
Supplement: Supplementary Table 4 — Correlation coefficients between riskScore and immune infiltrating cells (P < 0.05). [file Table_4.DOCX]

| immune | cor | pvalue | immune | cor | pvalue |
| --- | --- | --- | --- | --- | --- |
| Hematopoietic stem cell_XCELL | 0.3424 | 0.0000 | Myeloid dendritic cell_TIMER | 0.1312 | 0.0142 |
| stroma score_XCELL | 0.3150 | 0.0000 | Macrophage M2_QUANTISEQ | 0.1311 | 0.0142 |
| Macrophage_TIMER | 0.3120 | 0.0000 | Neutrophil_XCELL | 0.1268 | 0.0178 |
| Cancer associated fibroblast_MCPCOUNTER | 0.3048 | 0.0000 | Eosinophil_XCELL | 0.1112 | 0.0379 |
| Cancer associated fibroblast_EPIC | 0.2914 | 0.0000 | NK cell_QUANTISEQ | -0.1074 | 0.0449 |
| Cancer associated fibroblast_XCELL | 0.2902 | 0.0000 | T cell CD4+ (non-regulatory)_XCELL | -0.1083 | 0.0433 |
| Endothelial cell_XCELL | 0.2682 | 0.0000 | Plasmacytoid dendritic cell_XCELL | -0.1102 | 0.0396 |
| Endothelial cell_MCPCOUNTER | 0.2565 | 0.0000 | T cell CD4+ memory activated_CIBERSORT-ABS | -0.1129 | 0.0351 |
| Endothelial cell_EPIC | 0.2235 | 0.0000 | T cell CD4+ memory activated_CIBERSORT | -0.1226 | 0.0220 |
| Macrophage M2_CIBERSORT-ABS | 0.2219 | 0.0000 | B cell plasma_XCELL | -0.1321 | 0.0136 |
| Monocyte_CIBERSORT-ABS | 0.1953 | 0.0002 | T cell CD4+ Th1_XCELL | -0.1328 | 0.0130 |
| Myeloid dendritic cell_MCPCOUNTER | 0.1901 | 0.0004 | T cell CD8+ naive_XCELL | -0.1396 | 0.0090 |
| microenvironment score_XCELL | 0.1725 | 0.0012 | Class-switched memory B cell_XCELL | -0.1478 | 0.0057 |
| Macrophage_EPIC | 0.1643 | 0.0021 | T cell follicular helper_CIBERSORT | -0.1699 | 0.0014 |
| Macrophage M2_CIBERSORT | 0.1640 | 0.0021 | Common lymphoid progenitor_XCELL | -0.1767 | 0.0009 |
| Neutrophil_TIMER | 0.1546 | 0.0038 | T cell CD4+ Th2_XCELL | -0.1775 | 0.0009 |
| Monocyte_CIBERSORT | 0.1536 | 0.0040 | T cell CD4+ memory_XCELL | -0.1833 | 0.0006 |
| Monocyte_XCELL | 0.1483 | 0.0055 | T cell gamma delta_XCELL | -0.2106 | 0.0001 |
| Mast cell activated_CIBERSORT-ABS | 0.1378 | 0.0099 | uncharacterized cell_EPIC | -0.2889 | 0.0000 |
| Mast cell activated_CIBERSORT | 0.1331 | 0.0128 |  |  |  |
|  |  |  |  |  |  |
